# Isoprenylated Flavonoids with PTP1B Inhibition from *Macaranga denticulata*

**DOI:** 10.1007/s13659-015-0082-2

**Published:** 2016-01-20

**Authors:** Lai-Bin Zhang, Chun Lei, Li-Xin Gao, Jing-Ya Li, Jia Li, Ai-Jun Hou

**Affiliations:** Department of Pharmacognosy, School of Pharmacy, Fudan University, 826 Zhang Heng Road, Shanghai, 201203 People’s Republic of China; National Center for Drug Screening, State Key Laboratory of Drug Research, Shanghai Institute of Materia Medica, Chinese Academy of Sciences, 189 Guo Shou Jing Road, Shanghai, 201203 People’s Republic of China; Department of Chinese Medicine, School of Pharmacy, Xinxiang Medical University, 601 Jin Sui Road, Xinxiang, 453003 People’s Republic of China

**Keywords:** *Macaranga denticulata*, Euphorbiaceae, Isoprenylated flavonoids, Dentichalcones A–C, Protein tyrosine phosphatase 1B

## Abstract

**Abstract:**

Three new C-methylated and isoprenylated chalcone derivatives, dentichalcones A–C (**1**–**3**), together with six known compounds (**4**–**9**), were isolated from the twigs and leaves of *Macaranga denticulata*. Their structures were elucidated by spectroscopic analysis, including 1D, 2D NMR, and MS data. The known compounds, (2*E*)-1-(5,7-dihydroxy-2,2,6-trimethyl-*2H*-benzopyran-8-yl)-3-(4-methoxyphenyl)-2-propen-1-one (**4**), (2*E*)-1-(5,7-dihydroxy-2,2-dimethyl-2*H*-benzopyran-8-yl)-3-phenyl-2-propen-1-one (**5**), laxichalcone (**6**), macarangin (**7**), bonanniol A (**8**), and bonannione A (**9**), showed inhibitory activities against protein tyrosine phosphatase 1B (PTP1B) in vitro.

**Graphical Abstract:**

Three new C-methylated and isoprenylated chalcone derivatives, dentichalcones A–C (**1**–**3**), together with six known compounds, were isolated from the twigs and leaves of *Macaranga denticulata*. Some compounds showed inhibitory activities against PTP1B in vitro. 
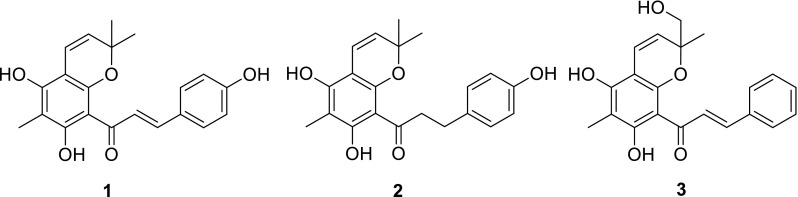

## Introduction

Isoprenylated flavonoids are a group of natural products with diverse structures and important bioactivities. A large number of new compounds have been isolated mainly from species of the Leguminosae, Moraceae, and Euphorbiaceae families, and some of the compounds showed antibacterial, antioxidant, anti-HIV, antidiabetic, and tyrosinase-inhibiting effects [1]. The genus *Macaranga* Thou. (Euphorbiaceae) comprises about 300 species mainly distributed in the tropical regions of Africa, Asia, Australia, and the Pacific islands [2]. The leaves of some *Macaranga* species have been used as folk medicine for the treatment of swellings, cuts, sores, boils, and bruises [3]. *Macaranga denticulata* (Bl.) Muell. Arg. is a tree with rich resources in Hainan and Xishuangbanna areas of China. Its roots have been used as traditional Chinese medicine against icteric hepatitis, eczema, and epigastric pain [4]. Previous phytochemical studies on this plant resulted in the isolation of isoprenylated flavonoids and diterpenylated flavonoids or stilbenes, some of which showed antioxidant, acetylcholinesterase-inhibiting, and antiangiogenic activities [5–7].

**Fig. 1:**
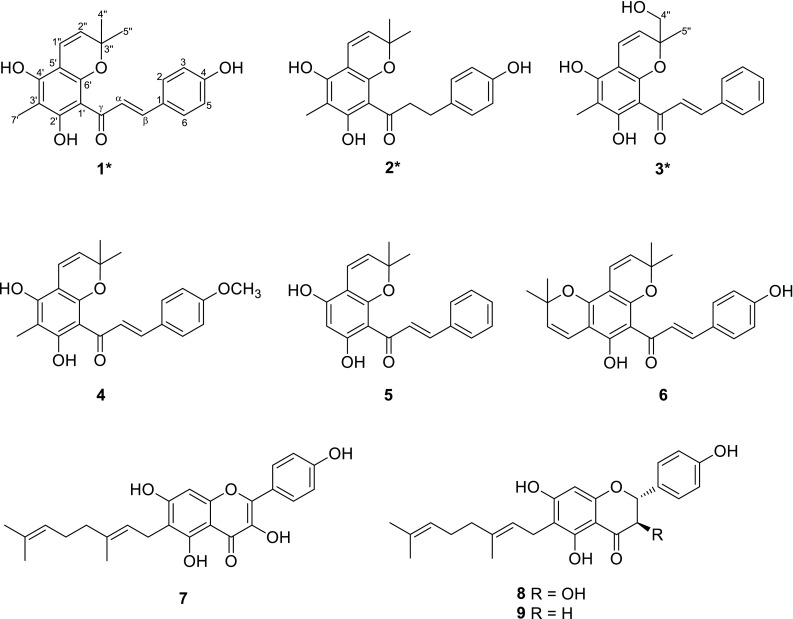
Structures of compounds **1–9**

Protein tyrosine phosphatase 1B (PTP1B) has been regarded as a promising target for treating type 2 diabetes and obesity [8]. Discovery of effective PTP1B inhibitors is one of our research interests [9–12], and a few of isoprenylated phenolics including flavonoids and Diels–Alder adducts were found to have significant PTP1B inhibitory effects [9, 10]. In our continuing search for natural PTP1B inhibitors from plants, chemical investigations of *M. denticulata* were carried out. Fractionation of the ethanol extract afforded nine isoprenylated flavonoids, including three new C-methylated and isoprenylated chalcones, dentichalcones A–C (**1–3**), together with six known compounds, (2*E*)-1-(5,7-dihydroxy-2,2,6-trimethyl-*2H*-benzopyran-8-yl)-3-(4-methoxyphenyl)-2-propen-1-one (**4**), (2*E*)-1-(5,7-dihydroxy-2,2-dimethyl-2*H*-benzopyran-8-yl)-3-phenyl-2-propen-1-one (**5**), laxichalcone (**6**), macarangin (**7**), bonanniol A (**8**), and bonannione A (**9**) (Fig. 1). The isolated compounds were tested in vitro for inhibition on PTP1B enzymatic activity. Compounds **4**–**9** showed significant inhibitory effects. This is the first report of C-methylated and isoprenylated chalcones from the genus *Macaranga*. Herein, we describe the structural elucidation and biological evaluation of these compounds.

## Results and Discussion

**Fig. 2:**
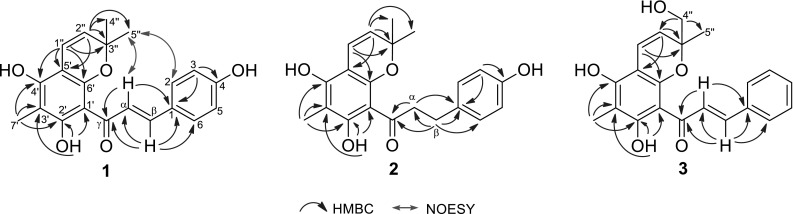
Selected HMBC and NOESY correlations of compounds **1–3**

Dentichalcone A (**1**) was assigned the molecular formula C_21_H_20_O_5_ by HREIMS with an *m/z* 352.1308 [M]^+^ (calcd for C_21_H_20_O_5_, 352.1311). The IR spectrum showed absorptions for OH (3417 cm^−1^), carbonyl (1625 cm^−1^), and aromatic (1605, 1514, and 1445 cm^−1^) moieties. The ^1^H NMR spectrum (Table 1) displayed a hydrogen-bonded hydroxyl signal at *δ*_H_ 14.62 (1H, s, OH-2′), two *trans*-coupled olefinic protons at *δ*_H_ 8.08 (1H, d, *J* = 15.6 Hz, H-α) and 7.76 (1H, d, *J* = 15.6 Hz, H-β), resonances of a 1,4-disubstituted benzene moiety at *δ*_H_ 7.61 (2H, d, *J* = 8.6 Hz, H-2, 6) and 6.95 (2H, d, *J* = 8.6 Hz, H-3, 5), a methyl group at *δ*_H_ 2.05 (3H, s, H_3_-7′), and signals of a 2,2-dimethylpyran ring at *δ*_H_ 6.70 (1H, d, *J* = 10.0 Hz, H-1″), 5.59 (1H, d, *J* = 10.0 Hz, H-2″), and 1.56 (6H, s, H_3_-4″, 5″). The ^13^C NMR spectrum (Table 1) exhibited 21 carbon signals: three methyls, eight *sp*^2^ methines, and ten quaternary carbons including a carbonyl, eight *sp*^2^, and one oxygenated *sp*^3^. These NMR spectroscopic data indicated that **1** was a chalcone derivative with an isoprenoid and a C-methyl group. By interpretation of the HMBC and NOESY spectra (Fig. 2), the structure of **1** was established. The HMBC cross-peaks of H-α/C-γ, C-1 and H-β/C-α, C-γ, C-1, C-6 verified the presence of chalcone skeleton. The hydroxyl group at *δ*_H_ 14.62 were assigned to OH-2′ by the HMBC correlations of OH-2′/C-1′, C-2′, C-3′. The methyl group at *δ*_H_ 2.05 (H_3_-7′) was located at C-3′ by the HMBC correlations of H_3_-7′/C-2′, C-3′, C-4′. The 2,2-dimethylpyran group was fused at C-5′ and C-6′, as deduced from the HMBC correlations of H-1″/C-4′, C-5′, C-6′ and H-2″/C-5′, together with the key NOESY correlations of H_3_-4″, 5″/H-α and H-2, 6. Thus, the structure of **1** was elucidated as (2*E*)-1-(5,7-dihydroxy-2,2,6-trimethyl-2*H*-benzopyran-8-yl)-3-(4-hydroxyphenyl)-2- propen-1-one and named dentichalcone A.

**Table 1:** ^1^H and ^13^C NMR spectroscopic data of compounds **1**–**3** (in acetone-*d*_6_)

Position	**1** ^a^		**2** ^a^		**3** ^a^	
	*δ*_C_	*δ*_H_ (*J* in Hz)	*δ*_C_	*δ*_H_ (*J* in Hz)	*δ*_C_	*δ*_H_ (*J* in Hz)
1	128.0		133.2		136.7	
2, 6	131.1	7.61, d (8.6)	130.0	7.10, d (8.3)	129.7	7.86, br d (7.3)
3, 5	116.9	6.95, d (8.6)	116.0	6.75, d (8.3)	129.5	7.44, m
4	160.7		156.4		130.8	7.44, m
α	125.1	8.08, d (15.6)	46.6	3.40, t (7.6)	129.0	8.54, d (15.5)
β	143.4	7.76, d (15.6)	30.4	2.89, t (7.6)	142.7	7.75, d (15.5)
γ	193.5		207.3		194.0	
1′	106.4		105.9		106.4	
2′	165.4		164.4		165.5	
3′	104.4		104.2		104.4	
4′	158.2		158.0		158.6	
5′	103.2		102.9		103.3	
6′	154.9		155.4		155.0	
7′	7.8	2.05, s^b^	7.7	2.05, s^b^	7.8	2.06, s^b^
1″	117.8	6.70, d (10.0)	117.6	6.65, d (10.0)	119.7	6.79, d (10.0)
2″	125.8	5.59, d (10.0)	125.8	5.53, d (10.0)	122.5	5.52, d (10.0)
3″	78.2		78.3		81.5	
4″	27.8	1.56, s	27.7	1.44, s	66.5	3.90, d (11.9) 3.61, d (11.9)
5″	27.8	1.56, s	27.7	1.44, s	22.7	1.57, s
OH-2′		14.62, s		14.10, s		14.52, s

^a^Data were measured at 400 MHz (^1^H) and 100 MHz (^13^C)

^b^Signals are overlapped

Dentichalcone B (**2**) was assigned the molecular formula C_21_H_22_O_5_ by HREIMS (*m/z* 354.1465 [M]^+^; calcd for C_21_H_22_O_5_, 354.1467). Comparison of its NMR spectroscopic data (Table 1) with those of **1** showed that **2** was a dihydrochalcone derivative of **1**. This was confirmed by some diagnostic signals at *δ*_H_ 3.40 (2H, t, *J* = 7.6 Hz, H_2_-α) and 2.89 (2H, t, *J* = 7.6 Hz, H_2_-β) and at *δ*_C_ 46.6 (C-α), and 30.4 (C-β), and 207.3 (C-γ) and by the HMBC correlations of H_2_-α/C-γ, C-1 and H_2_-β/C-α, C-γ, C-1, C-6 (Fig. 2). Thus, the structure of **2** was elucidated as 1-(5,7-dihydroxy-2,2,6-trimethyl-2*H*-benzopyran-8-yl)-3-(4-hydroxyphenyl)propan-1-one and named dentichalcone B.

Dentichalcone C (**3**) was assigned the molecular formula C_21_H_20_O_5_ by HRESIMS (*m/z* 351.1237 [M–H]^−^; calcd for C_21_H_19_O_5_, 351.1238). The ^1^H and ^13^C NMR spectra indicated the presence of a chalcone skeleton with a hydrogen-bonded hydroxyl and a methyl group, which was similar to that of **1**. Its NMR spectra also displayed signals of a monosubstituted benzene ring [*δ*_H_ 7.86 (2H, br d, *J* = 7.3 Hz, H-2, 6), 7.44 (3H, m, H-3, 4, 5); *δ*_C_ 136.7 (C-1), 130.8 (C-4), 129.7 (C-2, 6), and 129.5 (C-3, 5)] and resonances of a 2-hydroxymethyl-2-methylpyran moiety [*δ*_H_ 6.79 (1H, d, *J* = 10.0 Hz, H-1″), 5.52 (1H, d, *J* = 10.0 Hz, H-2″), 3.90 and 3.61 (each 1H, d, *J* = 11.9 Hz, H-4″a, b), 1.57 (3H, s, H_3_-5″); *δ*_C_ 119.7 (C-1″), 122.5 (C-2″), 81.5 (C-3″), 66.5 (C-4″), 22.7 (C-5″)]. The planar structure of **3** was further constructed by the HMBC spectrum (Fig. 2). The stereochemistry at C-3″ could not be assigned by the available data. Thus, the structure of **3** was elucidated as (2*E*)-1-[5,7-dihydroxy-2-(hydroxymethyl)-2,6-dimethyl-2*H*-benzopyran-8-yl]-3-phenyl-2-propen-1-one and named dentichalcone C.

The known compounds were identified as (2*E*)-1-(5,7-dihydroxy-2,2,6-trimethyl-*2H*-benzopyran-8-yl)-3-(4-methoxyphenyl)-2-propen-1-one (**4**) [13], (2*E*)-1-(5,7-dihydroxy-2,2-dimethyl-2*H*-benzopyran-8-yl)-3-phenyl-2-propen-1-one (**5**) [14], laxichalcone (**6**) [15], macarangin (**7**) [7], bonanniol A (**8**) [16], and bonannione A (**9**) [16] (Fig. 1) by comparison of their spectroscopic data with those reported. Compound **4** was a new natural product, which was previously reported as a synthetic molecule [13].

All the isolated compounds were tested in vitro for the inhibitory effects on PTP1B. Compounds **4**–**9** showed inhibition with IC_50_ values ranging from 14.0 ± 1.2 to 48.8 ± 5.5 μM (Table 2). Oleanolic acid, an effective natural PTP1B inhibitor [17], was used as the positive control (IC_50_ = 2.6 ± 0.6 μM).

**Table 2:** Inhibitory activities of compounds **4**–**9** against PTP1B

Compound	IC_50_ ± SD (µM)
**4**	48.8 ± 5.5
**5**	19.3 ± 1.4
**6**	20.7 ± 5.3
**7**	22.7 ± 4.6
**8**	15.2 ± 2.8
**9**	14.0 ± 1.2
Oleanolic acid	2.6 ± 0.6

In summary, this is the first report of C-methylated and isoprenylated chalcones from the genus *Macaranga*. This class of compounds is distributed limitedly in the family Euphorbiaceae, and only *Mallotus philippinensis* was reported to produce C-methylated or both C-methylated and isoprenylated chalcones [18–21]. The *Macaranga* and *Mallotus* genera are monophyletic sister groups in the family Euphorbiaceae, which show a remarkable resemblance in their phylogeny, habit, and geographical distribution [22]. The present study indicates that the two genera also have some similarity in their secondary metabolites. Furthermore, isoprenylated flavonoids as potent PTP1B inhibitors for the therapy of obesity and type 2 diabetes need further studies.

## Experimental Section

### General Experimental Procedures

Optical rotation was measured on a JASCO P-1030 digital polarimeter. UV spectra were recorded on a Hitachi U-2900 spectrophotometer. IR spectra were measured on a Nicolet Avatar-360 spectrometer with KBr pellets. NMR spectra were obtained on Varian Mercucy Plus 400 instruments. Chemical shifts were reported with TMS as internal standard or with respect to acetone-*d*_6_ (*δ*_H_ 2.04, *δ*_C_ 206.0 ppm). EIMS (70 eV) and HREIMS were recorded on an Agilent 5973N and a Waters Micromass GCT mass spectrometer, respectively. ESIMS and HRESIMS were performed on an Agilent 1100 LC/MSD and a Bruker Daltonics ApexIII mass spectrometer, respectively. Semi-preparative HPLC was performed on an Agilent 1200 (Agilent Technologies, Palo Alto, CA, USA) and a Sepax Amethyst C18 column (150 × 10 mm, 5 μm, Sepax Techologies, Inc., Newark, DE, USA), using a UV detector set at 210 nm. Column chromatography (CC) was performed on silica gel (200-300 mesh, Yantai Institute of Chemical Technology, Yantai, People’s Republic of China), Diaion HP-20 (Mitsubishi Chemical Co., Tokyo, Japan), and Sephadex LH-20 gel (GE Healthcare Amersham Biosciences, Uppsala, Sweden). Fractions were monitored by TLC analysis run on precoated silica gel GF254 plates (10–40 μm, Yantai Institute of Chemical Technology, Yantai, People’s Republic of China).

### Plant Material

The twigs and leaves of *M. denticulata* were collected in Hekou County, Yunnan Province, People’s Republic of China, in April 2011. The plant material was identified by Dr. Qin-Shi Zhao, Kunming Institute of Botany, Chinese Academy of Sciences, and a voucher specimen (TCM 11-04-15 Hou) has been deposited at the Herbarium of the Department of Pharmacognosy, School of Pharmacy, Fudan University.

### Extraction and Isolation

The milled, air-dried twigs and leaves of *M. denticulata* (5.0 kg) were percolated with 95 % EtOH at room temperature (60 L). The filtrate was evaporated under reduced pressure to give a residue (500 g), which was suspended in H_2_O and extracted with CH_2_Cl_2_ (4 × 1 L). The CH_2_Cl_2_ extract (140 g) was subjected to CC on Diaion HP-20 eluted with 90 % EtOH. The 90 % EtOH fraction (95 g) was separated by CC on silica gel eluted with a gradient of petroleum ether–EtOAc (1:0, 10:1, 5:1, 1:1, 1:2) to give fractions A–J. Fraction D was separated by CC on Sephadex LH-20 eluted with CHCl_3_–MeOH (1:1) to afford fractions D1–D4. Fraction D4 was chromatographed over silica gel eluted with a gradient of petroleum ether–Me_2_CO (10:1, 5:1) to afford fractions D4.1–D4.5. Fraction D4.3 was purified on a Sephadex LH-20 column eluted with MeOH to provide **4** (100 mg). Fraction E was chromatographed by silica gel eluted with a gradient of CH_2_Cl_2_–Me_2_CO (40:1, 2:1) to afford fractions E1–E8. Fraction E4 was separated on silica gel eluted with a gradient of petroleum ether–Me_2_CO (12:1, 10:1) to give fractions E4.1–E4.5. Fraction E4.5 was chromatographed by semi-preparative HPLC (CH_3_OH–H_2_O, 90:10, flow rate 1 mL/min) to afford **6** (10 mg). Fraction F was isolated by CC over Sephadex LH-20 eluted with CHCl_3_–MeOH (1:1) to afford fractions F1–F5. Fractions F2 and F4 were chromatographed over silica gel eluted with a gradient of petroleum ether–EtOAc (10:1, 2:1) to afford fractions F2.1–F2.6 and F4.1–F4.4, respectively. Fractions F2.2, F2.4, and F4.4 were purified by semi-preparative HPLC at flow rate 1 mL/min to afford **2** (4 mg; CH_3_OH–H_2_O, 83:17), **9** (10 mg; CH_3_OH–H_2_O, 90:10), and **7** (12 mg; CH_3_OH–H_2_O, 91:9), respectively. Fraction F4.2 was separated by CC on Sephadex LH-20 eluted with CH_3_OH to provide **1** (15 mg). Fraction G was separated by CC on silica gel eluted with a gradient of CH_2_Cl_2_–Me_2_CO (30:1, 2:1) to give fractions G1–G7. Fractions G2, G3, and G4 were chromatographed by semi-preparative HPLC at flow rate 1 mL/min to yield **5** (2 mg; CH_3_OH–H_2_O, 88:12), **3** (3 mg; CH_3_OH–H_2_O, 80:20), and **8** (12 mg; CH_3_OH–H_2_O, 88:12), respectively.

### Dentichalcone A (**1**)

Red, amorphous powder; UV (MeOH) *λ*_max_ (log ε) 229 (4.43), 244 (4.42) (sh), 289 (4.30), 368 (4.66) nm; IR (KBr) *ν*_max_ 3417, 2972, 2915, 1625, 1605, 1541, 1514, 1445, 1167, 830, 536 cm^−1^; ^1^H NMR and ^13^C NMR data, see Table 1; EIMS *m/z* 352 [M]^+^ (30), 337 (64), 217 (100), 91 (15), 77 (7); HREIMS *m/z* 352.1308 [M]^+^ (calcd for C_21_H_20_O_5_, 352.1311).

### Dentichalcone B (**2**)

Yellow, amorphous powder; UV (MeOH) *λ*_max_ (log ε) 222 (3.79), 284 (3.82) nm; IR (KBr) *ν*_max_ 3419, 2975, 2924, 1638, 1606, 1515, 1427, 1218, 1165, 1133, 827, 548 cm^−1^; ^1^H NMR and ^13^C NMR data, see Table 1; EIMS *m/z* 354 [M]^+^ (49), 339 (100), 233 (35), 219 (33), 191 (36), 107 (47), 91 (16), 77 (21), 65 (8), 43 (8); HREIMS *m/z* 354.1465 [M]^+^ (calcd for C_21_H_22_O_5_, 354.1467).

### Dentichalcone C (**3**)

Red, amorphous powder; [*α*]_D_^25^−32.9 (*c* 0.30, MeOH); UV (MeOH) *λ*_max_ (log ε) 224 (4.33), 240 (4.26), 287 (4.30), 346 (4.41) nm; IR (KBr) *ν*_max_ 3420, 2975, 2915, 1633, 1595, 1456, 1348, 1171, 1130, 701, 575 cm^−1^; ^1^H NMR and ^13^C NMR data, see Table 1; ESIMS *m/z* 351 [M–H]^−^; HRESIMS *m/z* 351.1237 [M–H]^−^ (calcd for C_21_H_19_O_5_, 351.1238).

### Assay of PTP1B Activity

The bioassay procedure was the same as that reported previously [10, 23]. The result of PTP1B inhibition was expressed as IC_50_, which was calculated with Prism 4 software (Graphpad, San Diego, CA).
